# What Does Dorsal Cortex Contribute to Perception?

**DOI:** 10.1162/opmi_a_00033

**Published:** 2020-08-01

**Authors:** Erez Freud, Marlene Behrmann, Jacqueline C. Snow

**Affiliations:** Department of Psychology and the Centre for Vision Research, York University; Department of Psychology and the Neuroscience Institute, Carnegie Mellon University; Department of Psychology, The University of Nevada Reno

**Keywords:** two visual pathways, object recognition, vision-for-perception, vision-for-action, affordance

## Abstract

According to the influential “Two Visual Pathways” hypothesis, the cortical visual system is segregated into two pathways, with the ventral, occipitotemporal pathway subserving object perception, and the dorsal, occipitoparietal pathway subserving the visuomotor control of action. However, growing evidence suggests that the dorsal pathway also plays a functional role in object perception. In the current article, we present evidence that the dorsal pathway contributes uniquely to the perception of a range of visuospatial attributes that are not redundant with representations in ventral cortex. We describe how dorsal cortex is recruited automatically during perception, even when no explicit visuomotor response is required. Importantly, we propose that dorsal cortex may selectively process visual attributes that can inform the perception of *potential* actions on objects and environments, and we consider plausible developmental and cognitive mechanisms that might give rise to these representations. As such, we consider whether naturalistic stimuli, such as real-world solid objects, might engage dorsal cortex more so than simplified or artificial stimuli such as images that do not afford action, and how the use of suboptimal stimuli might limit our understanding of the functional contribution of dorsal cortex to visual perception.

## TWO VISUAL PATHWAYS

The cortical visual system of human and nonhuman primates is anatomically segregated into two pathways, each of which is hierarchical in nature. These two pathways originate in early visual cortex and then diverge, with one projecting to the ventral cortex and extending to the lateral and inferior surfaces of the temporal lobes, and the other projecting to the dorsal cortex and extending to the posterior and superior aspects of the parietal lobes (Mishkin & Ungerleider, 1982).

Over the years, influential cognitive neuroscience theories have postulated that the two pathways are not only structurally dissociated from each other but are also functionally dissociated. Initially, it was suggested that the functional dissociation was based on the differential sensitivity of the pathways to specific visual attributes of the input. According to this division of labor, the “what” ventral pathway processes information about object properties (i.e., shape/geometry, and surface properties such as texture and color) and promotes “object vision,” while the “where” dorsal pathway encodes information about the location of the object in space and promotes “spatial vision” (Mishkin & Ungerleider, 1982; Mishkin, Ungerleider, & Macko, 1983). Roughly a decade later, this framework underwent revision with the recasting of the role of the pathways not based on the type of input information to which the pathway is responsive but, instead, by the contribution of each pathway to different functions or output requirements. On this reconceptualized view, the ventral pathway is responsible for vision-for-perception (i.e., the “what” pathway), while the dorsal pathway is responsible for vision-for-action (i.e., the “how” pathway) (Goodale & Milner, 1992). Just as was true for the what/where division of labor, the what/how distinction has been reinforced by decades of research with studies employing diverse methods, including neuroimaging, neuropsychology, and psychophysics (for a recent review, see Goodale & Milner, 2018).

In recent years, however, there is growing evidence that neither of these frameworks adequately captures the functional capabilities of each pathway. Critically, the evidence suggests that the dorsal pathway may not only be engaged in vision-for-action computations but might also play a role in object perception (Erlikhman, Caplovitz, Gurariy, Medina, & Snow, 2018; Freud, Plaut, & Behrmann, 2016), specifically in diverse perceptual functions such as 3D perception, localization of objects, and spatiotemporal integration.

In the current perspective, we review the evidence for the involvement of the dorsal pathway in the computation of four different visuospatial attributes, namely, depth, orientation, structure-from-motion, and shape, even under conditions that are independent of goal-directed actions. We also consider evidence consistent with the view that dorsal representations contribute uniquely to visual perception and are not simply redundant with information computed in ventral cortex. Furthermore, we discuss some aspects of visual development that might give rise to the emergence of these dorsal representations. We then argue that naturalistic visual inputs, such as real-world graspable solids, may engage the dorsal pathway more strongly than simplified or artificial stimuli such as pictures or computerized images, which are often used in vision science, and that the use of these suboptimal stimuli may have contributed to the underemphasis on the dorsal pathway in visual perception. Finally, we propose a new perspective on dorsal cortex function that centers on the premise that the potential for action offered by visual stimuli in naturalistic visual environments might shape the computations carried out by the dorsal pathway. These computations are then utilized to support action but can be used more extensively to support perception, as well.

## THE DORSAL PATHWAY PROCESSES VISUOSPATIAL ATTRIBUTES

**Figure 1.**
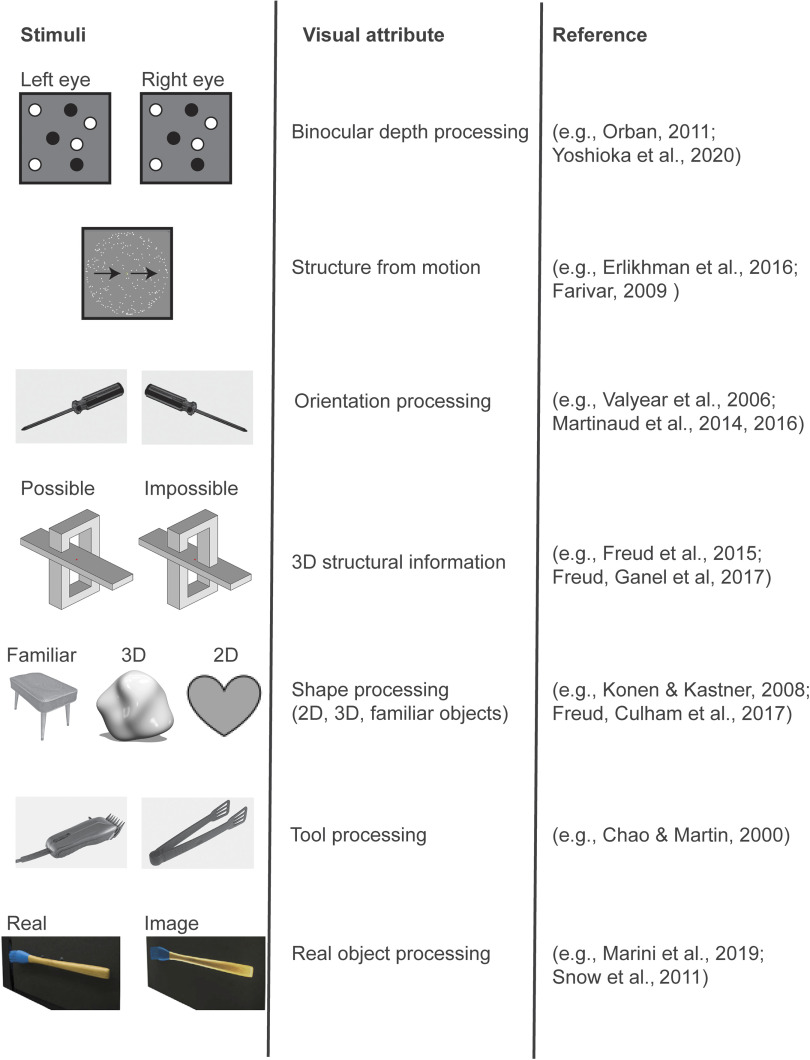
**The dorsal pathway is sensitive to different visual attributes.** Regions along the dorsal pathway process different visual attributes. The left column provides examples of the experimental stimuli that have been used. The middle column summarizes the visual attribute that is assumed to be processed by the dorsal pathway. The right column provides representative references.

In contrast with earlier characterizations of dorsal cortex that emphasize how visual inputs are used primarily to guide online control of action, research over the past two decades has revealed that the dorsal pathway is involved in processing visuospatial attributes of the visual input (for a discussion on the functional parcellation of the dorsal pathway, see Box 1). Importantly, this perceptual contribution of dorsal cortex is apparent even under conditions where no explicit visuomotor response is needed (for review, see Erlikhman et al., 2018; Freud et al., 2016). Below we describe some of the key findings from this literature (see Figure 1 for a summary).

**Box 1.**  Anatomical and functional parcellations of the dorsal pathway.

In this review, we often refer to “dorsal pathway representations.” However, it is important to note that the dorsal pathway territory includes significant portions of the occipital and the parietal lobes and is, therefore, composed of heterogeneous anatomical and functional subregions. These regions differ in their relative contribution to perceptual and visuomotor functions. The principal parcellation of the parietal lobe includes the postcentral gyrus, the superior parietal lobule, the inferior parietal lobule, the parietal operculum, and the intraparietal sulcus (IPS). Moreover, each of these regions can be further divided to smaller subregions (e.g., the IPS can be functionally subdivided to five or six regions based on retinotopic maps; Sereno et al., 1995; Swisher, Halko, Merabet, McMains, & Somers, 2007). Accordingly, previous studies have recognized the neural heterogeneity of the dorsal pathway and characterized the changes in neural representations of objects along this pathway.

Kravitz and colleagues (2011) have provided one of the most comprehensive accounts on the functional subdivision of the dorsal pathway. This account, which relies mostly on animal studies, suggests that the dorsal pathway is composed of three sub-pathways: The parieto–prefrontal pathway that supports spatial working memory, the parieto–premotor pathway that supports visually guided action, and the parieto–medial temporal pathway that primarily promotes spatial navigation. Importantly, the functional architecture of the three sub-pathways corresponds to the structural connections of each sub-pathway with other cortical structures.

The notion that the structural connectivity of regions along the dorsal pathway can dictate their relative contribution to perception and action is also mirrored in the idea that object representations in the dorsal pathway follow a posterior-anterior gradient of the dorsal pathway (Freud, Culham, Plaut, & Behrmann, 2017; Freud et al., 2016). In particular, posterior regions of the IPS are structurally and functionally coupled with the ventral pathway (Freud, Rosenthal, Ganel, & Avidan, 2015; Stepniewska, Cerkevich, & Kaas, 2015). Hence, those regions likely contribute more to perceptual functions, while more anterior regions, which are coupled with premotor, motor, and somatosensory cortices (Rushworth, Behrens, & Johansen-Berg, 2006), are more important for visuomotor transformations.

Lastly, different regions of parietal cortex are postulated to be involved in computations related to other cognitive functions that are not purely visual. This includes functions such as attention (Behrmann, Geng, & Shomstein, 2004; Buschman & Kastner, 2015; Greenberg et al., 2012), multisensory integration (Ghazanfar & Schroeder, 2006), working memory (D’Esposito & Postle, 2015), and numerical computation (Harvey, Klein, Petridou, & Dumoulin, 2013). One possibility is that the involvement of parietal cortex in these functions might reflect the extent to which spatial attributes are relevant for the specific processes, for example, spatial representations that underlie the conceptualization of a number line. The mapping between the nature of the information represented in dorsal cortex and the emergent function remains to be fully explicated.

### Three-Dimensional (3D) Depth Perception

The processing of depth information is imperative for the successful recognition of 3D objects, which, under naturalistic conditions, need to be recognized from different distances and viewpoints (Cox, 2014). Sensitivity of the dorsal pathway to this type of varying information, previously thought to be the sole purview of ventral cortex, has been documented in many studies with both human and nonhuman primates (for reviews, see Orban, 2011; Orban, Janssen,& Vogels, 2006). These studies have revealed responses to depth information induced from both monocular (Georgieva, Todd, Peeters, & Orban, 2008; Nelissen et al., 2009; Vanduffel et al., 2002) and binocular (i.e., disparity) cues (Georgieva, Peeters, Kolster, Todd, & Orban, 2009; Janssen, Vogels, & Orban, 2000; Yoshioka, Doi, Abdolrahmani, & Fujita, 2020), and have also uncovered responses to the global 3D structure of objects (Freud, Ganel, et al., 2017; Freud, Rosenthal, Ganel,& Avidan, 2015). Finally, activation in the dorsal pathway, as revealed using fMRI in humans, is observed in mental rotation (Gauthier et al., 2002), a process that is closely associated with computations related to 3D structural information. Similarly, the processing of configurally manipulated faces, which is related to volume derivation, elicits greater activation in the dorsal cortex than featured-based processing (Zachariou, Nikas, Safiullah, Gotts, & Ungerleider, 2016).

Nonetheless, it might be argued that sensitivity to depth information in the dorsal pathway is solely in the service of action and visuomotor transformations. However, recent behavioral evidence from healthy observers (Freud, Robinson, & Behrmann, 2018), studies of brain-damaged patients with visual agnosia (Freud, Ganel, et al., 2017), and nonhuman primates (Van Dromme, Premereur, Verhoef, Vanduffel, & Janssen, 2016), undermine this alternative hypothesis by showing that the dorsal pathway contributes to, and might even be necessary for, successful visuospatial perception (Berryhill, Fendrich, & Olson, 2009; Medina, Jax, & Coslett, 2020). In particular, Freud, Ganel, et al. (2017) showed that patients with visual agnosia following damage to ventral cortex (with extensive damage in some cases) were still able to derive the 3D structural information of objects and evinced preserved sensitivity of dorsal responses to this information. Consistently, reversible inactivation of the caudal IPS (i.e., a region in the dorsal pathway) in monkeys led to impaired 3D perception (Van Dromme et al., 2016). Visuospatial impairments have also been found in humans with lesions to the dorsal pathway (Berryhill et al., 2009; Medina et al., 2020), further supporting a causal relationship between dorsal cortex function and object perception.

### Orientation

Another visual attribute to which dorsal cortex shows sensitivity is the orientation of a stimulus and, of course, representing the orientation is necessary for grasping behavior. However, orientation is critical for perception too, for example, to distinguish between the enantiomorph letters “p” and “q,” which share geometric forms that are mirror images of each other. Damage to parietal cortex, for example, following a right occipitoparietal hematoma (Martinaud et al., 2014), or after a lesion affecting the posterior temporal gyrus and the inferior parietal lobule (Martinaud et al., 2016), results in persistent impairments in orientation and mirror-orientation processing. Likewise, neuroimaging findings from healthy individuals have demonstrated that the dorsal transverse occipital sulcus, but not the ventral cortex, is sensitive to the orientation of objects (Valyear, Culham, Sharif, Westwood, & Goodale, 2006) and to mirror transformations of scenes (Dilks, Julian, Kubilius, Spelke, & Kanwisher, 2011). These data reveal that the dorsal pathway is also involved in computing orientation for the purpose of perception, providing further support for the role of dorsal cortex in perceptual processing, independent of goal-directed actions.

### Structure-From-Motion

Motion is one of the most powerful cues for the perception of objects in dynamic, 3D, natural environments. Motion information can be used to compute the distance of an object, to integrate spatial information over time and to retrieve the 3D structure of objects, and regions of the dorsal pathway that are activated by 3D information are also sensitive to motion cues (for a detailed review, see Erlikhman et al., 2018).

The computations of Structure-from-Motion (SfM) are of particular interest, because they are thought to be the responsibility of the dorsal pathway and, therefore, independent from computations carried out by the ventral pathway (Farivar, 2009). SfM is achieved by exploiting the varying velocities of different points on the surface of the object. Movement-selective regions (middle temporal area, MT; and medial superior temporal area, MST) (Sugihara, Murakami, Shenoy, Andersen, & Komatsu, 2002), together with regions in posterior parietal cortex in humans (Erlikhman, Gurariy, Mruczek, & Caplovitz, 2016; Vanduffel et al., 2002), are involved in extracting SfM. Neuropsychological investigations offer converging evidence for the role of the dorsal pathway in the perception of SfM. For example, patients with occipitoparietal lesions, but not those with occipitotemporal lesions, are selectively impaired in perceiving 3D SfM and in detecting global motion patterns (Vaina, 1989) (see also preserved biological motion perception in individuals with ventral lesions, Gilaie-Dotan, Saygin, Lorenzi, Rees, & Behrmann, 2015). Importantly, this impairment in SfM perception did not reflect a general deficit in motion processing, as the patients with occipitoparietal lesions were still able to detect local motion.

### Shape

Shape processing, standardly attributed to the ventral pathway, is perhaps one of the most intriguing test cases for the role of dorsal cortex in perception. Shape processing provides the foundation for a diverse range of perceptual and visuomotor behaviors and involves higher-level integration of multiple visual cues. Whereas early neuroimaging research identified shape-selectivity in the lateral occipital and posterior fusiform cortices within the ventral occipitotemporal (vOT) pathway (Grill-Spector et al., 1998; Malach et al., 1995), later studies also uncovered shape selectivity in parietal cortex. These initial observations of shape selectivity in dorsal cortex were attributed to the involvement of attention (Kourtzi & Kanwisher, 2000), or to the association of certain types of visual stimuli such as tools (versus non-tool objects) with visuomotor actions (Chao & Martin, 2000; Mruczek, von Loga, & Kastner, 2013). However, more recent evidence has revealed shape selectivity in multiple regions of the dorsal pathway for non-tool objects (Bracci & Op de Beeck, 2016; Freud, Culham, Plaut, & Behrmann, 2017), for novel objects with no semantic associations (Freud et al., 2015; Konen & Kastner, 2008), and even for 2D line-drawings of objects or basic shapes (Konen & Kastner, 2008; Sereno & Maunsell, 1998).

**Figure 2.**
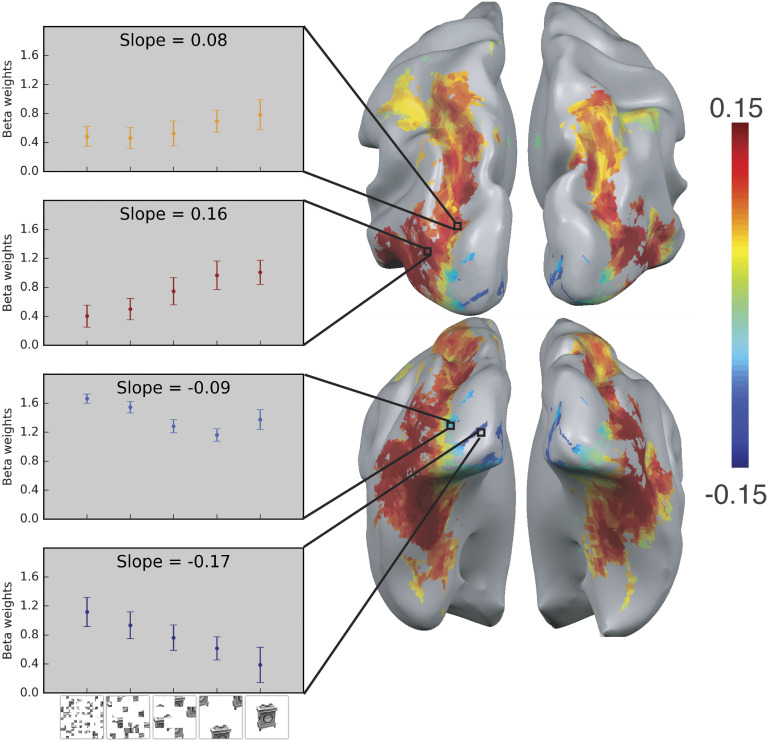
**Shape processing along the dorsal pathway.** Shape sensitivity along both pathways was explored using fMRI by utilizing a parametric scrambling manipulation in which shape information is degraded gradually (left panel, bottom row). The activation profile of four representative clusters (10 voxels each) is plotted and the color of the bars reflects the slope value of each cluster (left panel). Shape sensitivity is projected on an inflated brain from a superior view (upper right panel) and from a posterior-inferior view (lower right panel). Warm colors signify voxels that are shape sensitive, with activation increasing as a function of object coherence. Conversely, cold colors reflect low shape sensitivity (negative slopes) or greater sensitivity for scrambled than intact images. Notably, shape sensitivity was observed not only along the ventral pathway but also in the dorsal pathway (Freud, Culham, et al., 2017).

Many studies have been devoted to elucidating the nature of shape representations derived by the dorsal-response profile and a number of important conclusions have been reached. First, dorsal shape selectivity is not uniform across parietal cortex (Kravitz et al., 2011), but rather, follows a representational gradient, with greater shape sensitivity in more posterior regions compared with more anterior regions that are tuned to visuomotor aspects (Freud, Culham, Plaut, & Behrmann, 2017; see Figure 2 and Box 1). Second, although shape representations in the dorsal pathway can be modulated by input from (Mahon, Kumar, & Almeida, 2013) or by damage confined to (Freud & Behrmann, 2020) the ventral pathway, these dorsal representations are not just epiphenomenal or a byproduct resulting from cascaded signals from the ventral pathway. Studies using high–temporal resolution methods, like ERP, MEG, and single-cell recording, have revealed that dorsal shape-selectivity signals precede those of ventral shape signals (Collins, Freud, Kainerstorfer, Cao, & Behrmann, 2019; Lehky & Sereno, 2007; Liu, Wang, Zhou, Ding, & Luo, 2017), making it unlikely that dorsal activation is merely projected from ventral shape computations. Also, recent developmental neuroimaging findings in normally developing children ranging in age from 6 to 21 years, reveals that dorsal cortex matures earlier than ventral cortex, suggesting that representations in dorsal cortex are unlikely to be constrained a priori by ventral signals (Ciesielski et al., 2019). Finally, based on human fMRI data, the representational similarity between shape-selective responses in the two visual pathways can be greater than the similarity within each of the pathways, further emphasizing the importance of both pathways in shape processing (Freud, Culham, Plaut, & Behrmann, 2017). Nevertheless, unlike ventral representations, dorsal representations are more adaptive than ventral representations, and they are subject to task demands. For example, fMRI classification accuracies of object category from dorsal region decreased when participants completed a task that was unrelated to the objects, while classification accuracies from ventral regions were more invariant of the task (Vaziri-Pashkam & Xu, 2017, 2018; Xu, 2018).

### What Gives Rise to Dorsal Pathway Representations?

Some stimulus attributes processed in dorsal cortex (reviewed above), such as SfM, appear to be unique and under the sole purview of the dorsal visual pathway, whereas others, such as orientation and shape, are represented by both dorsal and ventral pathways. One might then ask what the functional contribution of dorsal representations is to visual perception and to what extent is this contribution distinct from ventral cortex? Does dorsal cortex contribute to visual perception by processing the elements considered above or are there additional contributions? Even though there is overlap in the visual attributes processed by the two pathways, dorsal and ventral cortex may still contribute in different ways (Konen & Kastner, 2008). For example, dorsal cortex might use these inputs to guide perception in a qualitatively different fashion than ventral cortex. In other words, even though largely similar visual inputs are utilized in both visual streams, they may still serve different perceptual purposes.

One possibility is that dorsal cortex processes certain visual attributes because the attributes offer useful physical constraints for *potential* actions with objects and environments. Specifically, the visual attributes reviewed above, including 3D depth, SfM, and geometric shape, may be selectively extracted for further processing by dorsal cortex because they specify regularities in the natural world that can be exploited to inform and constrain the perception of environmental opportunities for action. Real-world environments consist of a complex 3D array of tangible solids of different shapes, sizes, and weights, and of course, observers move around the environment, thereby changing the relative egocentric distances between the body and other solids. Notably, the idea that the potential for action can promote dorsal cortex processing is also consistent with neuropsychological and behavioral studies that documented enhanced perceptual abilities for objects that are placed near the hands and therefore are more relevant for actions (Brockmole, Davoli, Abrams, & Witt, 2013; Cosman & Vecera, 2010; Gozli, West, & Pratt, 2012).

Importantly, this perspective of dorsal cortex function bears some resemblance to the “vision-for-action” and “vision-for-perception” framework (Goodale & Milner, 1992) in that it emphasizes the different output requirements of the two pathways. But the key difference lies in the distinction that the dorsal pathway is recruited not only during “in-the-moment” actions but also during visual perception for detecting potential opportunities for action with objects and environments, whether or not an action is planned or executed. This view dovetails with “ecological” frameworks of perception that emphasize that visual perception serves to facilitate action in real-world contexts (Gibson, 1979), and evolutionary arguments that the human brain has presumably evolved to allow us to perceive and interact with real objects and environments (Cisek & Kalaska, 2010; Heft, 2013).

Such a “potential action” mechanism for dorsal contributions to visual perception, in which representations are modulated by attributes such as 3D shape, viewpoint, egocentric distance, and physical size, also contrasts with theoretical accounts of the ventral processing stream in which responses to objects remain predominantly stable or “invariant,” despite changes in viewing conditions that alter object appearance but not identity (Bar, 2004; Grill-Spector, Kourtzi, & Kanwisher, 2001; Grill-Spector & Malach, 2004; Kravitz, Vinson, & Baker, 2008; Logothetis & Sheinberg, 1996). Because subtle changes in visual parameters can have a dramatic influence on behavior, it seems advantageous to have perceptual responses or representations in dorsal cortex that are largely stable (i.e., not invariant) when a stimulus conveys visual cues that signify concrete physical attributes (Holler, Fabbri, & Snow, 2020). For example, knowing the absolute egocentric distance of an object from the body is critical for determining whether the object lies within reach (Jeannerod, 1981) and for pre-shaping the hand for grasping (Castiello, 2005; Jeannerod, 1986; Smeets & Brenner, 1999). However, these conjectures contrast with evidence for “content rich,” complex representations of behaviorally relevant objects in dorsal cortex that appear to be represented in a highly abstract manner (Jeong & Xu, 2016; Xu, 2018). For example, images of task-relevant faces and cars are represented invariantly in human parietal cortex despite changes in visual parameters such as background scene, viewpoint or size (Jeong & Xu, 2016). However, as we outline later (see Dorsal Cortex May Play a Unique Role in Perceiving Action-Relevant Stimuli and Environments), it remains to be seen whether dorsal representations respond more or less invariantly depending upon the format in which the stimulus is displayed (Holler et al., 2020; Snow et al., 2011), and whether or not the stimulus classes investigated are items that would typically be grasped and manipulated with the hands. It also remains an empirical question as to whether shape images (i.e., a picture of a baseball or a mug) would stimulate dorsal cortex more strongly if they were displayed so that the retinal size matched the real-world size, or indicated that the object was an appropriate size for manipulation. Although recent neuropsychological evidence suggests that manipulations of image size alone may not be sufficient to engage dorsal cortex (Holler, Behrmann, & Snow, 2019), it remains to be seen whether additional cues to size, such as stereoscopic depth or background scene context, could engage dorsal cortex more effectively.

Powerful links between perception and action emerge over time, and over the course of development, in parallel with repeated experience with objects in the natural environment (Bertenthal, 1996; Fan et al., 2020; Lockman & Kahrs, 2017). These encounters may be the source of such bidirectional perception–action interactions. One compelling demonstration of how goal-directed actions shape perception comes from disruptions to visual perception in human observers who actively or passively explored the natural environment while wearing vision-adjusting prism lenses (Held & Bossom, 1961; Held & Mikaelian, 1964). The need for coupling perception and action over development is even more clearly demonstrated in the early “kitten-carousel” study by Held and Hein (1963) in which two kittens reared in darkness from birth were harnessed to opposite ends of a horizontal arm in a carousel. Both animals received the same visual stimulation but one kitten was harnessed with its feet touching the ground, allowing it to walk actively around the carousel, while the other was harnessed with its feet on a platform and was moved passively by the “self-moving” kitten partner. Later, when the kittens were removed from the carousel, the self-moving kitten demonstrated normal visual perception, while the passively moving kitten showed abnormalities in visually guided paw placements, as well as in depth and distance perception. An analogous finding from humans comes from the study of individuals whose sight was restored surgically (Held et al., 2011). The key question was whether previously blind individuals would be able to recognize visually an object that had only been perceived tactilely. The individuals failed to recognize the object visually immediately after their sight was restored, but crossmodal mappings developed rapidly, and after only 5 days of natural real-world visual experience their crossmodal matching ability was close to ceiling (Held et al., 2011). This suggests that real-world action can tune or recalibrate this intersensory matching process rather rapidly. (See Box 2 for an elaborated discussion on the development of dorsal object representations.)

**Box 2.**  Developmental emergence of dorsal object representations.

One source of data that might inform our understanding of the relative contribution of dorsal object representations and its coupling to action comes from studies of the developmental emergence of dorsal cortex and motor development. The critical hypothesis is that if dorsal object representations are configured or constrained by action, then one might expect that the dorsal pathway would not represent object perception until *after* motor systems are mature enough to permit interaction with objects (see section What Gives Rise to Dorsal Pathway Representations? for related questions addressed in adults with movement impairments). The early emergence of reaching behaviors is consistent with the notion that action such as reaching not only influences action perception (Cannon, Woodward, Gredebäck, von Hofsten, & Turek, 2012; Sommerville, Woodward, & Needham, 2005) but also influences visual perception in important ways, especially during early development (Needham & Libertus, 2011).

Although there has not been a study conducted across ages (either longitudinal or cross-sectional) that is specifically and systematically focused on the chronological emergence of perception vis-à-vis action, there do exist data that can shed light on this matter. For example, several studies have argued that prehension in infants can significantly guide the infants’ learning about objects and their properties. In one such study, infants were divided into two groups, one of which was able to pick up objects using active “sticky” mittens and the other of which simply watched their parents interact with the objects (Libertus & Needham, 2010). Only the infants from the former condition showed changes in their visual exploration of agents and objects in a live setting, leading to the conclusion that early action can influence reaching as well as visual behavior. A similar finding was obtained in a later study in which infants played with toys. One group of infants again was fitted with sticky mittens while a second group had passive “nonsticky” mittens. In advance of this, infants were given a teether to explore. Only infants who were in the active sticky mittens condition, but not those in the passive condition, demonstrated an increase in looking at and exploring the teether more after than before training (Needham, Wiesen, Hejazi, Libertus, & Christopher, 2017). And, relatedly, young infants either manipulated objects actively themselves or received objects passively presented to them (Needham & Libertus, 2011). Spontaneous orienting toward faces and objects was only evident in the former and not in the latter group, revealing a potential link between manual engagement and the development of orienting toward faces.

These findings argue for the influence of early actions on perception. There are also studies offering evidence for slightly later development of visual perception of 3D objects, which would allow for earlier emergence of action skills. For example, infants only show sensitivity to 3D object completion at 6 months of age (with girls at only 9 months of age) (Soska & Johnson 2008, 2013). Lastly, there are some data showing that even if action does not precede perception, these skills emerge concurrently. For example, object completion abilities emerge in conjunction with developing motor skills, most notably, independent sitting and visual-manual exploration, as these two skills predict looking behavior in an incomplete 3D object task (Soska, Adolph, & Johnson, 2010). Together, these studies argue in favor of action-based representations helping configure perceptual representations.

Nevertheless, differentiating the contribution of action entirely independent from that of perception is not trivial. For example, in one study, infants aged 5 to 15 months viewed different sets of balls; one set was rigid in structure and required a full-hand power grasp, and the other set was nonrigid and could be acquired with full precision grasp just with fingertips (Barrett, Traupman, & Needham, 2008). Reaching movement was evaluated prior to contact with the ball and the results showed very different forms of prehension for the two sets of balls. This led to the conclusion that visuomotor constraints that are present early in development led to the appropriate differentiation of the grasp. In other words, the reaching (or action alone) was not sufficient and visual information about the balls contributed to alterations in reaching. In sum, reaching definitive conclusions about the emerging sequence of action versus perception and their influence on each other is not always possible, and while action certainly appears to modulate perception, the converse may likely be true as well.

One particularly persuasive piece of evidence for the role of dorsal cortex in object perception, entirely independent of action, would be findings that show that dorsal cortex is engaged in object perception in patients who are paralyzed or who never had use of their hands. Such data would support the view that object perception is not solely contingent on the presence of action or on action-relevant affordances (Gibson, 1979). Although we have not been able to find such data, one finding worth noting is that patients born without hands (dysplasia) and who use their feet instead of hands, appear to have typical motor selectivity (albeit in inferior parietal lobule, rather than in motor cortex) leading to the suggestion that high-level representations are effector-invariant (and perhaps affordance based) (Striem-Amit, Vannuscorps, & Caramazza, 2018; Vannuscorps & Caramazza, 2016). Relatedly, in these individuals, it is also the case that motor cortex or other regions (like the inferior parietal lobule) show motor selectivity of the compensatory effector (Striem-Amit et al., 2018; Vannuscorps, Wurm, Striem-Amit, & Caramazza, 2019). A second set of related studies with dysplasic participants has reported the finding of normal hand- and tool-selectivity and their overlap in ventral cortex, reflecting functional organization in ventral cortex even in the absence of sensorimotor experience (Striem-Amit, Vannuscorps, & Caramazza, 2017). As evident, although the above findings are of interest, the most compelling data of dorsal-based object perception in the absence of any action constraint have not been reported as yet. Such a finding would further cement the claim of independence of dorsal object representations.

We have suggested above that dorsal cortex selectively processes certain visual attributes because of the constraints they impose on perception in the service of action, and that this perception–action relationship appears to follow a global posterior-to-anterior visual-to-motor gradient, with more posterior regions such as caudal intraparietal area (CIP) devoted to processing features such as 3D curvature, and more anterior regions such as anterior intraparietal sulcus (aIPS) involved in computing the metrics required for motor actions with objects (Culham & Valyear, 2006; Fabbri, Stubbs, Cusack, & Culham, 2016; Shmuelof & Zohary, 2005; Stark & Zohary, 2008; also see Freud et al., 2016). Through this lens, the notion of perception in the service of action could perhaps be characterized as a Bayesian prior that is scaled by object size in the context of body space. Behavioral and neurophysiological findings point to the existence of a network of areas along occipitoparietal cortex, including cortical areas in humans such as V3A, V7/IPS0, and IPS, that selectively process object attributes that are important for action (Erlikhman et al., 2018). Notably, many of these regions of parietal cortex represent actions within the co-ordinate reference frame of specific effectors, such as the eyes, hands, or arms (Andersen & Buneo, 2002; Andersen, Snyder, Bradley, & Xing, 1997; Colby & Goldberg, 1999; Gallivan & Culham, 2015).

There also appears to be an emphasis within dorsal cortex on the representation of egocentric distance and peripersonal space—the area around the body that is within reach of the arms (Duhamel, Bremmer, Hamed, & Graf, 1997; Galati et al., 2000; Galletti, Battaglini, & Fattori, 1993; Rizzolatti, Fadiga, Gallese, & Fogassi, 1996; Vallar et al., 1999). Importantly, one region, the superior occipitotemporal cortex (SPOC), selectively responds to graspable objects that are within reach of the observer, but not when they lie outside of reach, even when no physical grasping action is required toward the object (Cavina-Pratesi et al., 2018; Gallivan, Cavina-Pratesi, & Culham, 2009). Similarly, some dorsal regions tuned to stereo-depth show greater sensitivity to manipulations of egocentric versus allocentric distance (Neggers, Van der Lubbe, Ramsey, & Postma, 2006; Neri, Bridge, & Heeger, 2004). Convergent evidence for the involvement of dorsal cortex in egocentric distance perception comes from neuropsychological patients with lesions of parietal cortex (Brain, 1941). Some patients show selective impairments in estimating the distance from the body to nearby objects, or placing objects at specific distances from the body (Berryhill et al., 2009), although such deficits could also reflect more fundamental impairments in spatial or orientation processing that are limited to the egocentric reference frame.

### Dorsal Cortex May Play a Unique Role in Perceiving Action-Relevant Stimuli and Environments.

Despite the fact that, in everyday life, humans predominantly interact with solid objects in real-world 3D environments, visual perception has classically been studied in experimental psychology and neuroscience using impoverished stimuli in the form of static two-dimensional (2D) images of objects. One problem with this approach, particularly for understanding the functional role of dorsal cortex and its potential role in coding potential actions with objects and environments, is that 2D images convey little, if any, information about the types of visual attributes that dorsal cortex is uniquely sensitive to, such as 3D shape and depth, SfM, egocentric distance, and real-world size. For example, from the perspective of an observer looking at a planar image of an object on a computer screen, the distance to the projection surface is known, but not the distance to the depicted object. The real-world size of the stimuli used in studies of visual perception is further obscured when objects are presented as pictorial cutouts abstracted from their corresponding background, and when the retinal extent of items that are typically large in the world (i.e., a horse) is matched with items that are orders of magnitude smaller (i.e., a butterfly) (Bracci & Op de Beeck, 2016; Cichy, Kriegeskorte, Jozwik, van den Bosch, & Charest, 2019; Konkle & Oliva, 2012; Kriegeskorte, Mur, & Bandettini, 2008).

**Figure 3.**
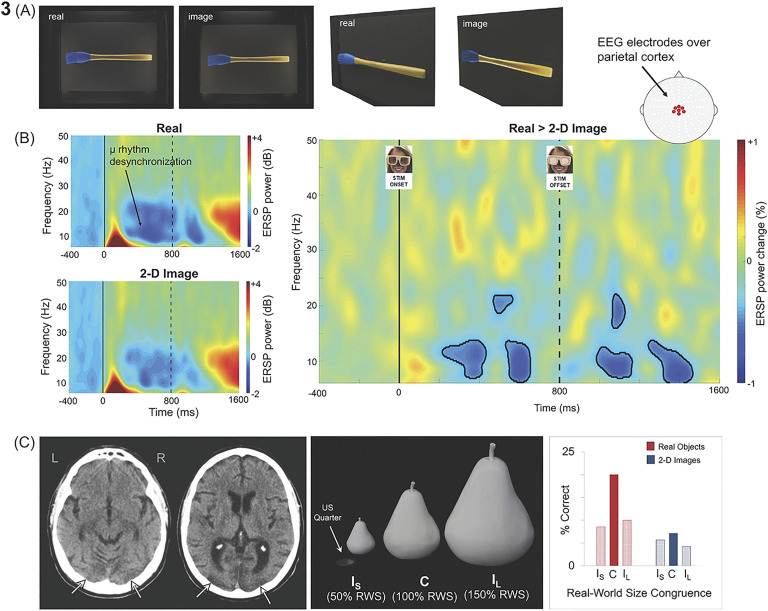
**The processing of real objects** (A). Stimuli used in an EEG study by Marini et al. (2019) to investigate whether real-world objects elicit distinct visuomotor brain dynamics compared to matched 2D images of the same items. In the study, EEG recordings were collected while observers viewed real tools and 2D images of tools. When viewed from the front, the stimuli were matched closely for apparent size, distance, background, illumination, and color (left panel). For illustrative purposes, the same stimuli are shown (right panel) from an oblique perspective. (B). Real objects increased the strength and duration of activation in brain networks involved in automatic action planning relative to images of objects. In the study described, Marini et al. (2019) decomposed EEG signals to reveal desynchronization of the *μ* (“mu”) rhythm (8–13 Hz)—a frequency-specific change associated with the automatic transformation of visual object information into action representations. Left panels illustrate event-related spectral perturbation (ERSP) power recorded from central electrodes positioned over parietal cortex, shown separately for real objects (upper panel) and 2D images (lower panel). Although both stimulus formats elicited *μ* rhythm desynchronization, this effect was stronger and more sustained for real objects in comparison to 2D images (right panel, black outlines demarcate areas of statistical significance). The stronger *μ* desynchronization for the real objects (versus images) was apparent during stimulus presentation and persisted after stimulus offset (demarcated by dashed vertical line) (Marini et al., 2019). (C). Real-world size coding of solid objects in a patient with visual agnosia. JW, a patient with bilateral lesions to occipitotemporal cortex (left panel) was presented with solid objects and 2D computerized images of the same items that were scaled to be incongruent smaller (Is), congruent (C), or incongruent larger (IL) than typical real-world size (middle panel). While recognition of images was extremely poor, real object recognition was surprisingly preserved, but only when physical size matched real-world size (right panel) (Holler et al., 2019).

Interestingly, convergent evidence from studies using a range of empirical approaches, including behavioral psychophysics, fMRI, EEG, and neuropsychology, has begun to highlight fundamental differences in the way real objects and computerized images are processed during perception (Gerhard, Culham, & Schwarzer, 2016; Holler et al., 2019, 2020; Squires, Macdonald, Culham, & Snow, 2016), memory (Snow, Skiba, Coleman, & Berryhill, 2014), attention (Gomez et al., 2017), and decision-making (Romero, Compton, Yang, & Snow, 2018). Importantly, these effects may be driven by the selective recruitment of dorsal cortex during perceptual processing. For example, passively viewing real objects triggers stronger and more prolonged automatic motor preparation signals than does viewing matched images of the same objects, as measured by high-density EEG over parietal cortex, particularly in the hemisphere contralateral to the dominant hand (Marini et al., 2019) (see Figure 3A–Figure 3B).

Compellingly, evidence from neuropsychology further suggests that dorsal cortex is critically involved in perceiving object shape and size, specifically for real objects (but not images). Holler et al. (2019) examined object recognition in patients with severe visual agnosia resulting from bilateral lesions of shape processing areas in ventral cortex (see Figure 3C). Although the patients were severely impaired in their ability to recognize 2D images of objects, they showed a striking preservation in their ability to recognize real-world exemplars of the same stimuli. Critically, however, the recognition advantage shown by the patients for the real objects was only apparent when the physical size of the (real) objects was consistent with the typical real-world size. Recognition of objects whose physical size deviated from real-world size was severely impaired and similar to 2D images. Analogous manipulations of the visual size of 2D computerized images did not modulate recognition performance. These findings are all consistent with the claim that visual representations in dorsal cortex may be constrained by real-world properties of objects and it is these representations that then primarily serve action as well as perception.

## CONCLUSIONS

In summary, we have reviewed evidence that dorsal cortex is particularly sensitive to a range of visuospatial attributes including SfM, 3D geometric shape, orientation, and size. We have also advanced the idea that dorsal cortex selectively processes these visual attributes because of the constraints they impose for perception in the service of action, and that, while these constraints are predominantly relevant in the context of real-world objects and environments, they can also mediate vision for perception. This characterization of the role of dorsal cortex for perception contrasts with traditional frameworks that ascribed a more monolithic role that centered upon action alone, as well as with conceptualizations of the role of ventral cortex, in which object processing operates predominantly to achieve invariant responses to objects to stabilize conscious perception despite changing visual conditions.

One of the reasons why it has been difficult to characterize fully the role of dorsal cortex is that working with more naturalistic conditions and real objects in the laboratory presents a number of practical and empirical challenges that are not encountered when using image displays (Romero & Snow, 2019). Whether or not dorsal cortex is particularly tuned to processing actionable solid objects or is also tuned for perception independent of action, and whether dorsal cortex responds only or specifically to visual properties that are relevant to action, are questions of outstanding importance that require careful investigation. To further advance our understanding of the two cortical pathways, future experiments should include a wider range of required behaviors and stimuli.

## FUNDING INFORMATION

JCS, National Eye Institute, Award ID: R01EY026701, the National Science Foundation (NSF), Award ID: 1632849; and the Clinical Translational Research Infrastructure Network, Award ID: 17-746Q-UNR-PG53-00. MB, National Eye Institute, Award ID: RO1EY027018. EF, Natural Sciences and Engineering Research Council of Canada (501100000038), Award ID: NA, and Vision Science to Applications (VISTA) program funded by the Canada First Research Excellence Fund (CFREF, 2016–2023) (EF).

## AUTHOR CONTRIBUTIONS

EF: Conceptualization: Equal; Visualization: Equal; Writing - Original Draft: Equal; Writing - Review & Editing: Equal. MB: Conceptualization: Equal; Writing - Review & Editing: Equal. JCS: Conceptualization: Equal; Visualization: Equal; Writing - Original Draft: Equal; Writing - Review & Editing: Equal.
